# Supplementary material for: Sonic hedgehog-heat shock protein 90β axis promotes the development of nonalcoholic steatohepatitis in mice
Source: Nat Commun. 2024 Feb 12;15:1280. doi: 10.1038/s41467-024-45520-8 (PMC10859387; doi:10.1038/s41467-024-45520-8)
Supplement: Supplementary file 1 — Supplementary Information [file 41467_2024_45520_MOESM1_ESM.pdf]

Title: Sonic hedgehog-heat shock protein 90β axis promotes the development of nonalcoholic steatohepatitis in mice

Authors: Weitao Zhang[1,2], Junfeng Lu[3], Lianshun Feng[2], Hanyue Xue[2], Shiyang Shen[2], Shuiqing Lai[4], PingPing Li[5], Ping Li[2], Jian Kuang[4], Zhiwei Yang[6]* and Xiaojun Xu[1,2]*

Affiliations:

1. Department of Pharmacy, The Fourth Affiliated Hospital, Zhejiang University School of Medicine, Yiwu, Zhejiang, China; Center for Innovative Traditional Chinese Medicine Target and New Drug Research, International Institutes of Medicine, Zhejiang University, Yiwu, Zhejiang, China.

2. State Key Laboratory of Natural Medicines, China Pharmaceutical University, 210009, Nanjing, Jiangsu, China.

3. First Department of Liver Disease, Beijing You'An Hospital, Capital Medical University, Beijing 100069, China.

4. Guangdong Provincial People's Hospital, Guangdong Academy of Medical Sciences, Guangzhou, Guangdong, China.

5. State Key Laboratory of Bioactive Substance and Function of Natural Medicines, Institute of Materia Medica, Chinese Academy of Medical Sciences & Peking Union Medical College, Diabetes Research Center of Chinese Academy of Medical Sciences, Beijing, 100050, China.

6. Institute of Laboratory Animal Science, Chinese Academy of Medical Sciences (CAMS) & Peking Union Medical Collage (PUMC), Beijing, 100021, PR China.

Corresponding author:

Prof. Xiaojun Xu, Department of Pharmacy, The Fourth Affiliated Hospital, Zhejiang University School of Medicine, Yiwu, Zhejiang, China; Center for Innovative Traditional Chinese Medicine Target and New Drug Research, International Institutes of Medicine, Zhejiang University, Yiwu, Zhejiang, China. E-mail: xiaojunxu@zju.edu.cn.

Prof. Zhiwei Yang, Institute of Laboratory Animal Science, Chinese Academy of Medical Sciences (CAMS) & Peking Union Medical Collage (PUMC)

E-mail: yangzhiwei@cnilas.pumc.edu.cn.

Supplementary figures

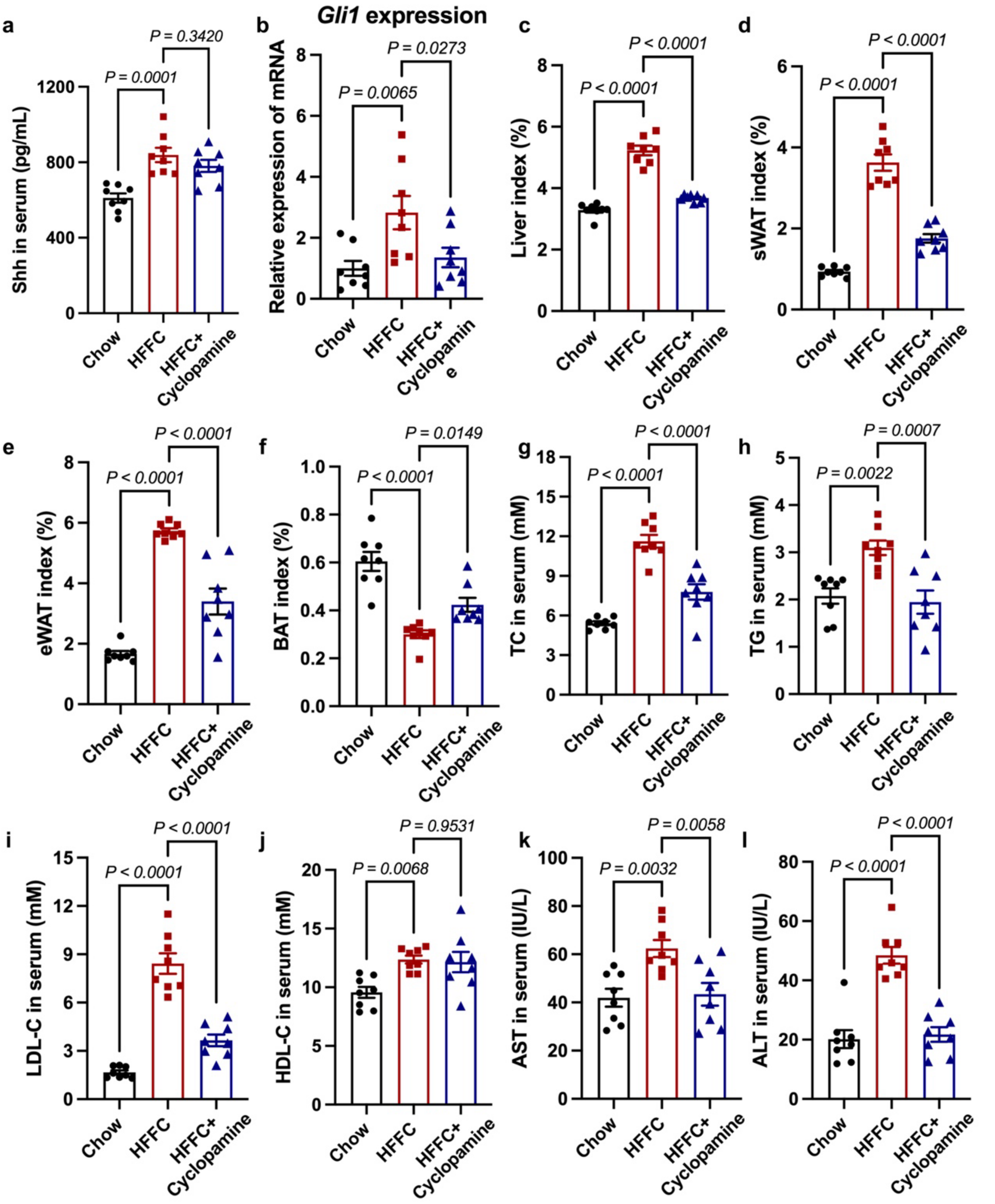

Supplementary Figure 1 Cyclopamine protects mice from HFFC diet-induced fatty liver. a-l Serum Shh (**a**), relative *Gli1* gene expression (**b**), Liver index (**c**), eWAT index (**d**), sWAT index (**e**), BAT index (**f**), serum TC (**g**), serum TG (**h**), serum LDL-c (**i**), serum HDL-c (**j**), Serum ALT (**k**), and serum AST (**l**) of mice on a normal chow or HFFC diet administrated with vehicle or cyclopamine (25 mg/kg) (*n* = 8 mice per group). Data are presented as mean ± SEM. *n* = 8 mice per group. Source data are provided in the Source Data file.

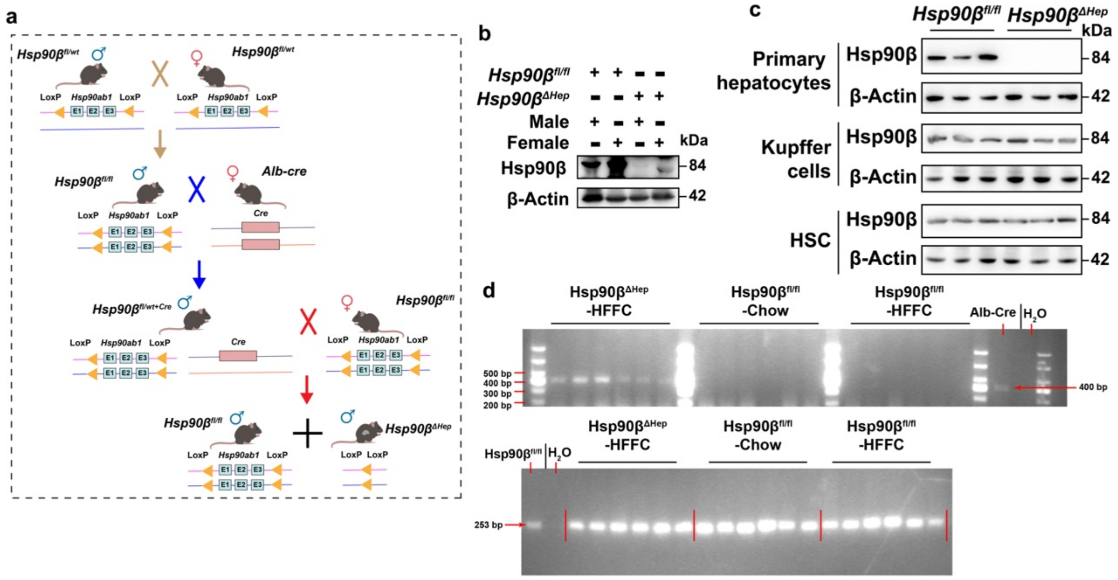

Supplementary Figure 2 Generation and verification of *Hsp90β^{ΔHep}* mice. a Schematic illustration of the mating strategy to generate hepatocellular Hsp90β knockout mice (*Hsp90β^{ΔHep}*) by crossing *Hsp90β$^{fl/fl}$* and *Alb-Cre* mice. **b** Western blot analysis of Hsp90β in the liver tissues of male or female *Hsp90β$^{fl/fl}$* or *Hsp90β^{ΔHep}* mice. **c** Western blot analysis of primary hepatocyte, Kupffer cells and HSCs from *Hsp90β$^{fl/fl}$* and *Hsp90β^{ΔHep}* mice. **d** PCR confirmation of *Hsp90β$^{fl/fl}$* and *Hsp90β^{ΔHep}* mice using mouse tail DNA. Source data are provided in the Source Data file.

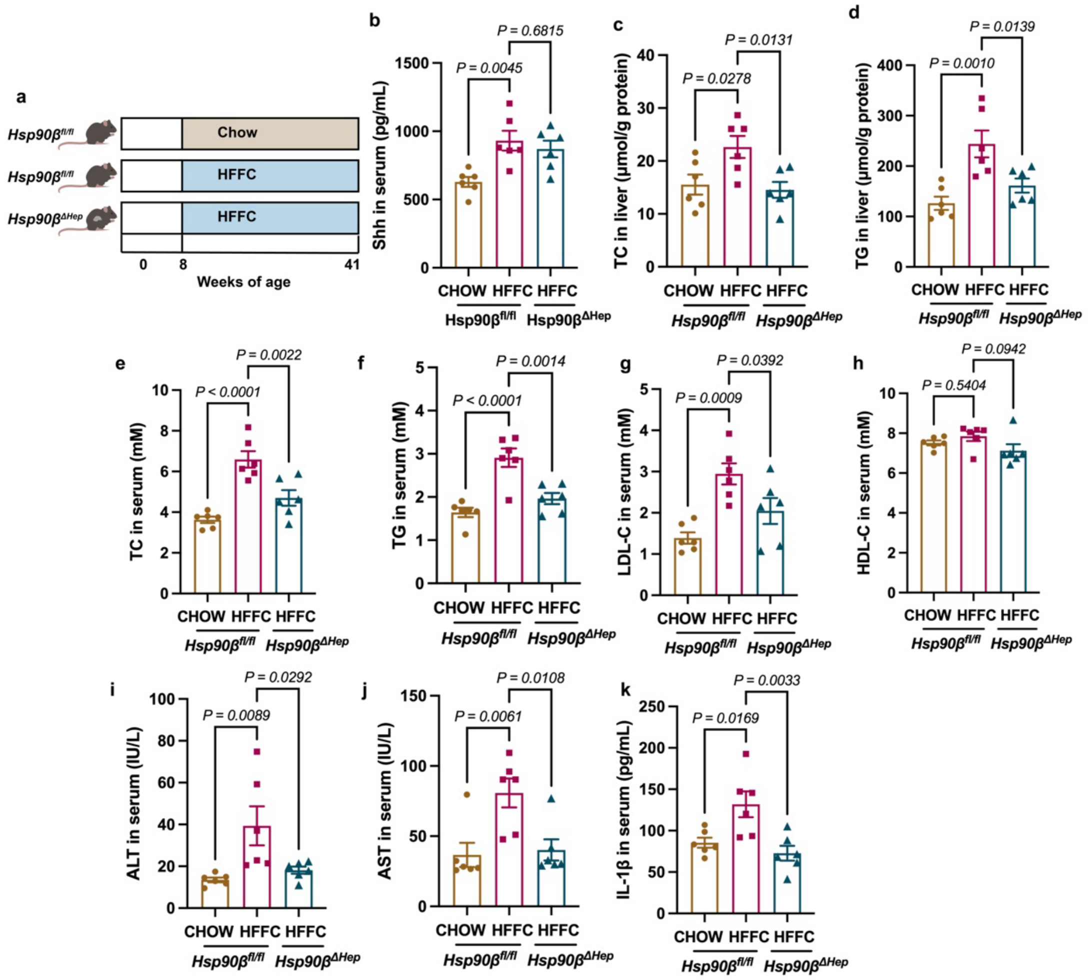

Supplementary Figure 3 Hepatic deletion of *Hsp90β* ameliorates diet-induced NASH. a Experimental scheme. **b-k** Serum Shh (**b**), TC in liver (**c**), TG in liver (**d**), serum TC (**e**), serum TG (**f**), serum LDL-c (**g**), and serum HDL-c (**h**), Serum ALT (**i**), serum AST (**j**) and Serum IL-1β levels (**k**) of *Hsp90β*$^{fl/fl}$ and *Hsp90β*^{ΔHep} mice on a normal chow or HFFC diet (*n* = 6 mice per group). Data are presented as mean ± SEM. *n* = 6 mice per group. Source data are provided in the Source Data file.

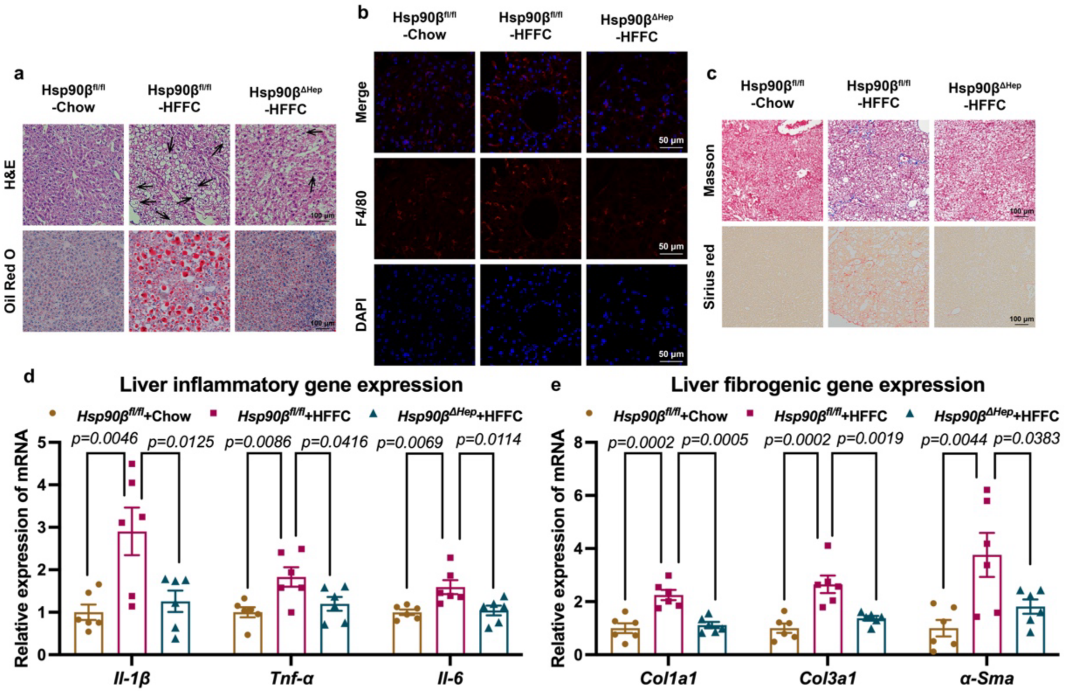

Supplementary Figure 4 Hepatic deletion of *Hsp90β* prevents HFFC-induced NASH pathologies. **a** Representative H&E stained FFPE liver sections and ORO stained frozen liver sections. Each arrow indicated hepatocyte ballooning. **b** Representative immunofluorescence F4/80 stained liver sections **c** Representative Masson and Sirius red stained liver sections **d-e** Expression of inflammatory (**d**) and fibrogenic (**e**) genes in livers of $Hsp90\beta^{fl/fl}$ and $Hsp90\beta^{\Delta Hep}$ mice on a normal chow or HFFC diet (n = 6 mice per group). Data are presented as mean ± SEM. n = 6 mice per group. Source data are provided in the Source Data file.

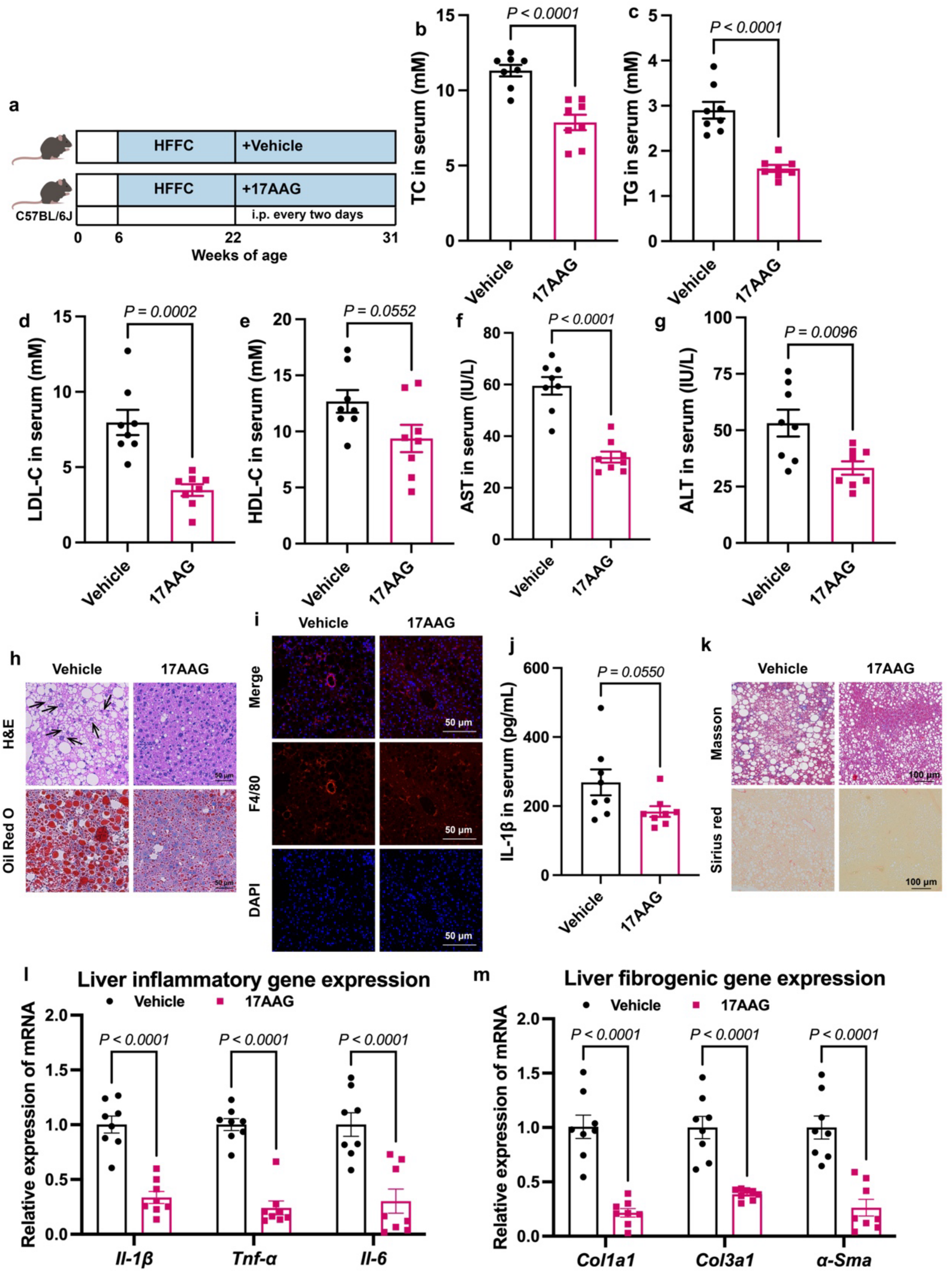

Supplementary Figure 5 17AAG exerts protective effects against HFFC-induced NASH phenotype. **a** Experimental scheme. **b-g** serum TC (**b**), serum TG (**c**), serum LDL-c (**d**), serum HDL-c (**e**), Serum AST (**f**), and serum ALT (**g**). **h** Representative H&E stained FFPE liver sections and ORO stained frozen liver sections. Each arrow indicated hepatocyte ballooning. **i** Representative immunofluorescence F4/80 stained liver sections. **j** Serum IL-1β levels. **k** Representative masson and sirius red stained liver sections. **l-m** Expression of inflammatory (**l**), and fibrogenic (**m**) genes in livers of mice on a HFFC diet administrated with vehicle or 17AAG (1 mg/kg) (n = 8 mice per group). Data are presented as mean ±

SEM. n = 8 mice per group. Source data are provided in the Source Data file.

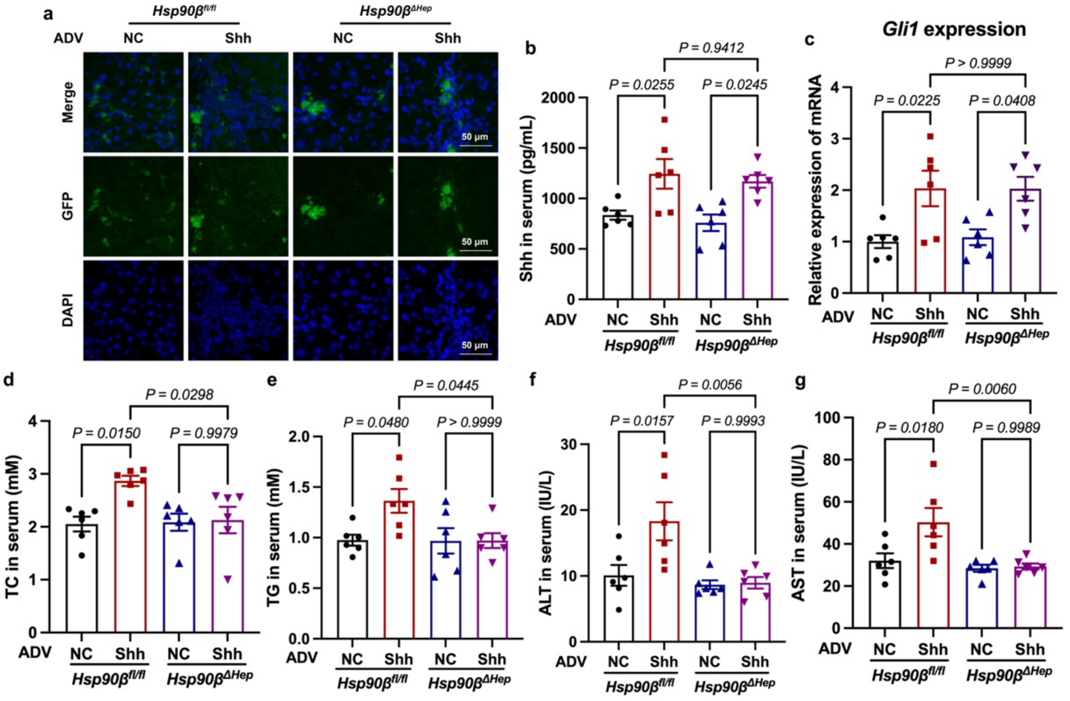

Supplementary Figure 6 Shh overexpression in *Hsp90β^{ΔHep}* mice. a Representative immunofluorescence GFP stained liver sections of *Hsp90β$^{fl/fl}$* and *Hsp90β^{ΔHep}* mice injected with ADV–NC or ADV-Shh on HFFC diet (n = 6 mice per group). **b-g** serum SHH (**b**), *Gli1* gene expression (**c**), serum TC (**d**), serum TG (**e**), serum ALT (**f**), and serum AST (**g**) of *Hsp90β$^{fl/fl}$* and *Hsp90β^{ΔHep}* mice injected with ADV–NC or ADV-Shh on HFFC diet (n = 6 mice per group). Data are presented as mean ± SEM. n = 6 mice per group. Source data are provided in the Source Data file.

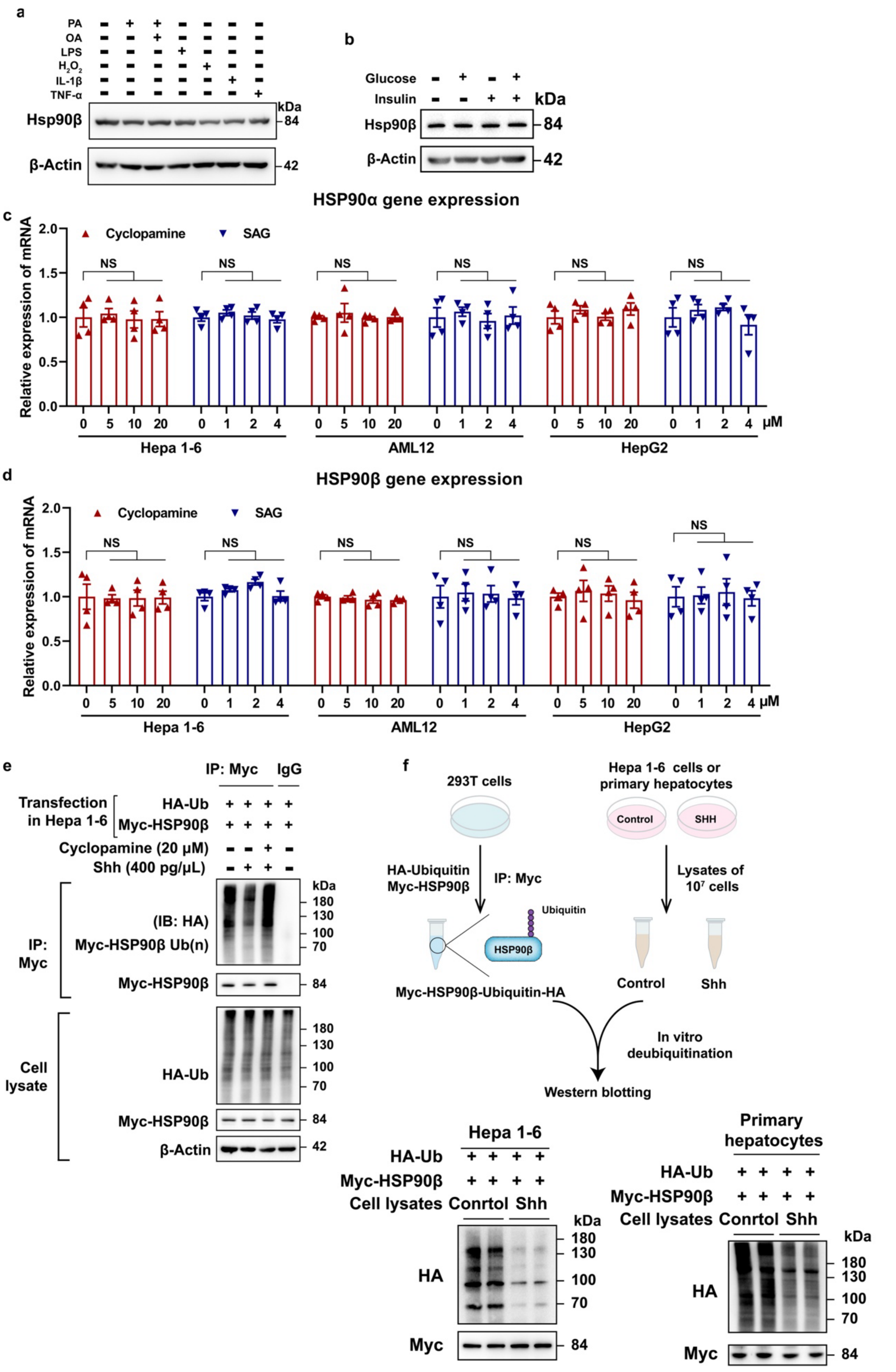

Supplementary Figure 7 Shh mediates Hsp90β protein levels by deubiquitylation. a-b (**a**) Western blot analysis of Hepa 1-6 cells treated with 400 μM oleic acid (OA), 200 μM Palmitic acid (PA), 1 μg/mL

lipopolysaccharide (LPS), 10 μM H_2O_2, 25 ng/mL IL-1β, 5 ng/mL TNF-α, (**b**) 30 mM glucose, or 100 nM insulin. **c-d** (**c**) *Hsp90α* and (**d**) *Hsp90β* gene expression in Hepa 1-6, AML12 and HepG2 cells treated with cyclopamine or SAG. **e** HSP90β ubiquitylation assays were performed in primary hepatocytes treated with 400 pg/μL Shh with or without 20 μM cyclopamine for 24 hours after plasmid Myc-HSP90β and HA-ubiquitin transfection. The cell lysates were incubated with immunoprecipitated Myc and then immunoblotted with anti-HA to reveal the ubiquitylation levels. **f** Schematic representation of *in vitro* deubiquitylation assay. The ubiquitylated Myc-HSP90β was immunoprecipitated from HEK293T cells, incubated with the lysates from Hepa 1-6 cells treated with or without 400 pg/μl Shh, and analyzed by immunoblotting. Data are presented as mean ± SEM. n = 4 independent experiments per group. Source data are provided in the Source Data file.

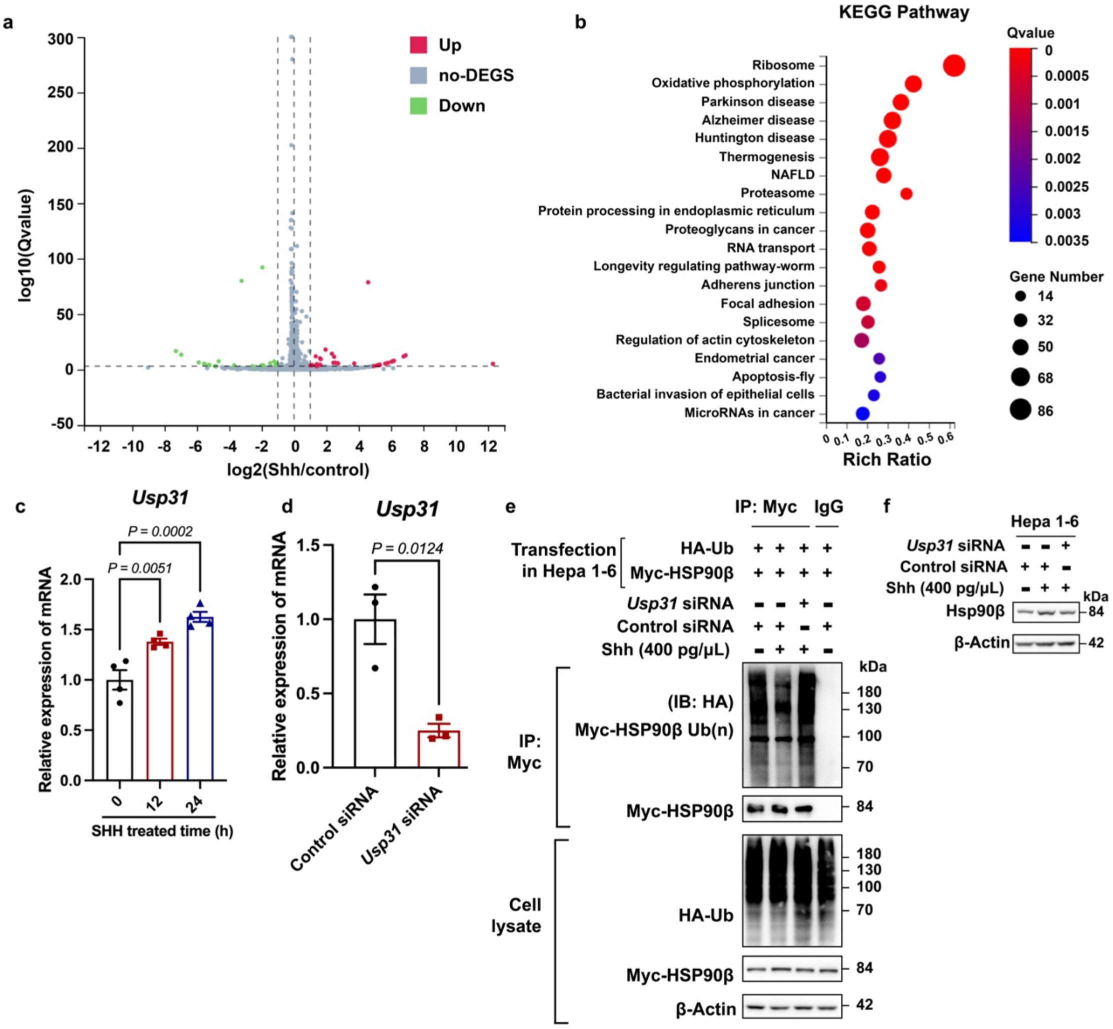

Supplementary Figure 8 Usp31 is a DUB of Hsp90β. a Heatmap analysis of RNA-seq data from Hepa 1-6 treated with water or Shh. Color represents log2 fold changes between Shh and control. **b** KEGG

pathway of RNA-seq data from Hepa 1-6 treated with water or Shh. **c** *Usp31* gene expression in Hepa 1-6 cells treated with 400 pg/μl Shh for 12 and 24 hours. *n* = 4 independent experiments per group. **d** The knockdown efficiency of RNAi targeting *Usp31* assessed by qRT-PCR. *n* = 3 independent experiments per group. **e** HSP90β ubiquitylation assays were performed in Hepa 1-6 cells treated with 400 pg/μL Shh after Usp31 knocked down and plasmid Myc-HSP90β and HA-ubiquitin transfection. The cell lysates were incubated with immunoprecipitated Myc and then immunoblotted with anti-HA to reveal the ubiquitylation levels. **f** Hepa 1-6 cells were transfectied with *Usp31* RNAi for 48 hours, then cells were treated with 400 pg/μL Shh for 24 hours, Hsp90β protein level was analyzed by immunoblotting. Data are presented as mean ± SEM. Source data are provided in the Source Data file.

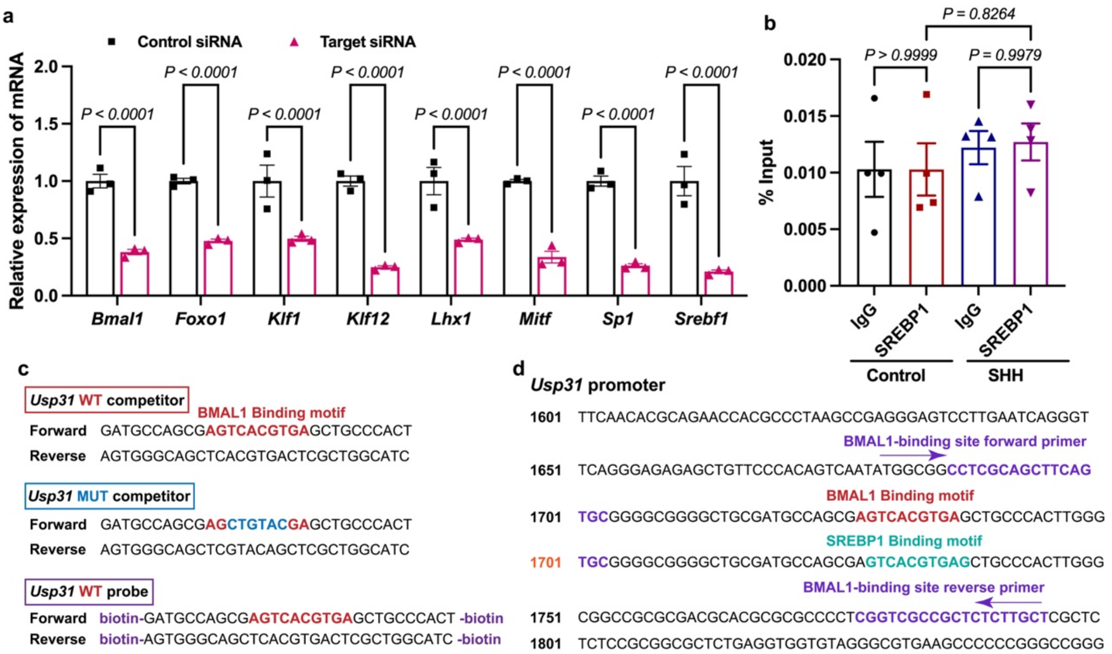

Supplementary Figure 9 BMAL1 binds the *Usp31* promoter. a The knockdown efficiency of 8 TFs was assessed by qRT-PCR. **b** In the presence/absence of Shh, the binding of SREBP1 to the *Usp31* promoter region was analyzed by ChIP analysis in Hepa 1-6 cells. **c** Putative BMAL1 and *Usp31* binding sites, EMSA probe in the *Usp31* promoter region. **d** Putative BMAL1 and *Usp31* binding sites, ChIP primers in the *Usp31* promoter region. Source data are provided in the Source Data file.

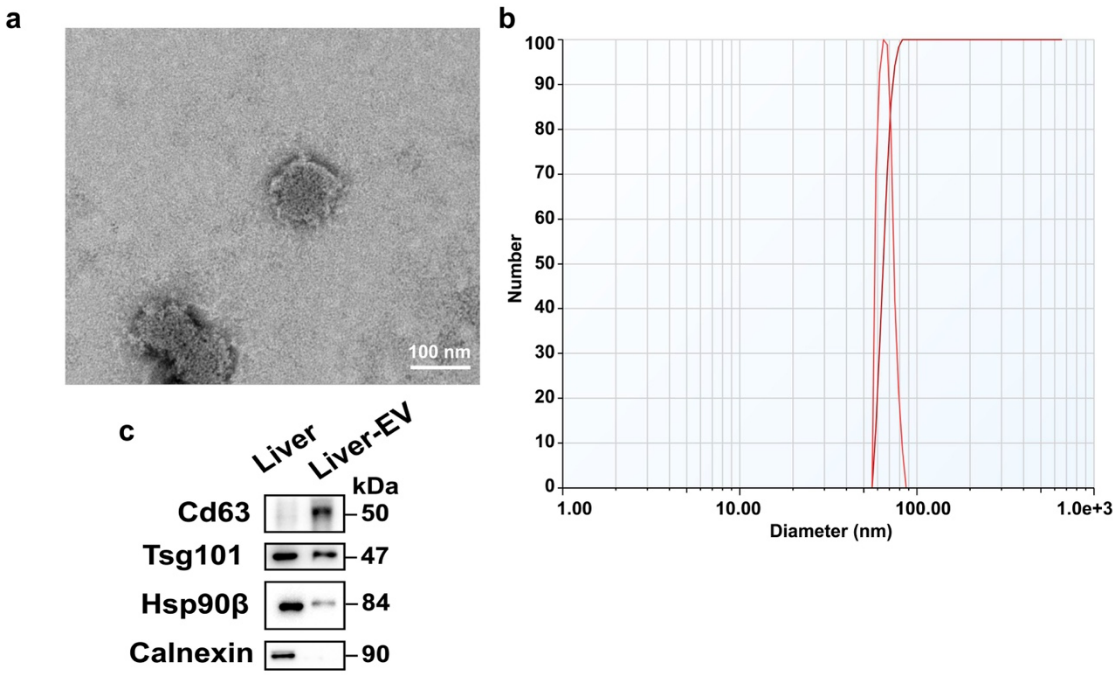

Supplementary Figure 10 Characteristics of exosomes. a Representative Transmission Electron Microscope (TEM) image of EVs derived from livers. **b** Particle diameter of EVs derived from livers. **c** Representative bands of Western blot for EV markers (Cd63, Tsg101, and Hsp90β) as well as the negative marker (Calnexin) in EVs derived from livers. Source data are provided in the Source Data file.

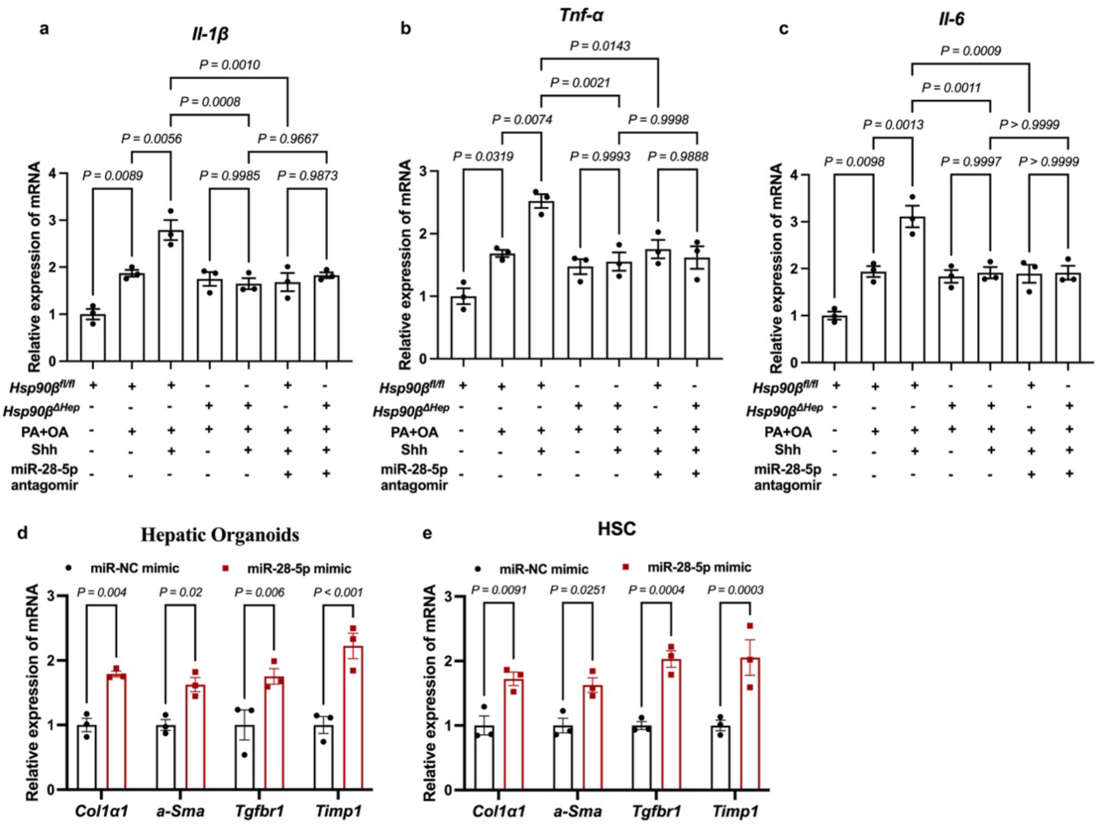

Supplementary Figure 11 Shh induces inflammation in organoids. a-c Organoids were treated as

indicated and the expression of inflammatory genes was analyzed by qRT-PCR (n = 3 independent experiments per group). **d** Fibrogenic genes expression in hepatic organoids when administrated with miR-NC mimic and miR-28-5p mimic (n = 3 independent experiments per group). **e** Fibrogenic genes expression in HSCs when administrated with miR-NC mimic and miR-28-5p mimic (n = 3 independent experiments per group). Data are presented as mean ± SEM. Source data are provided in the Source Data file.

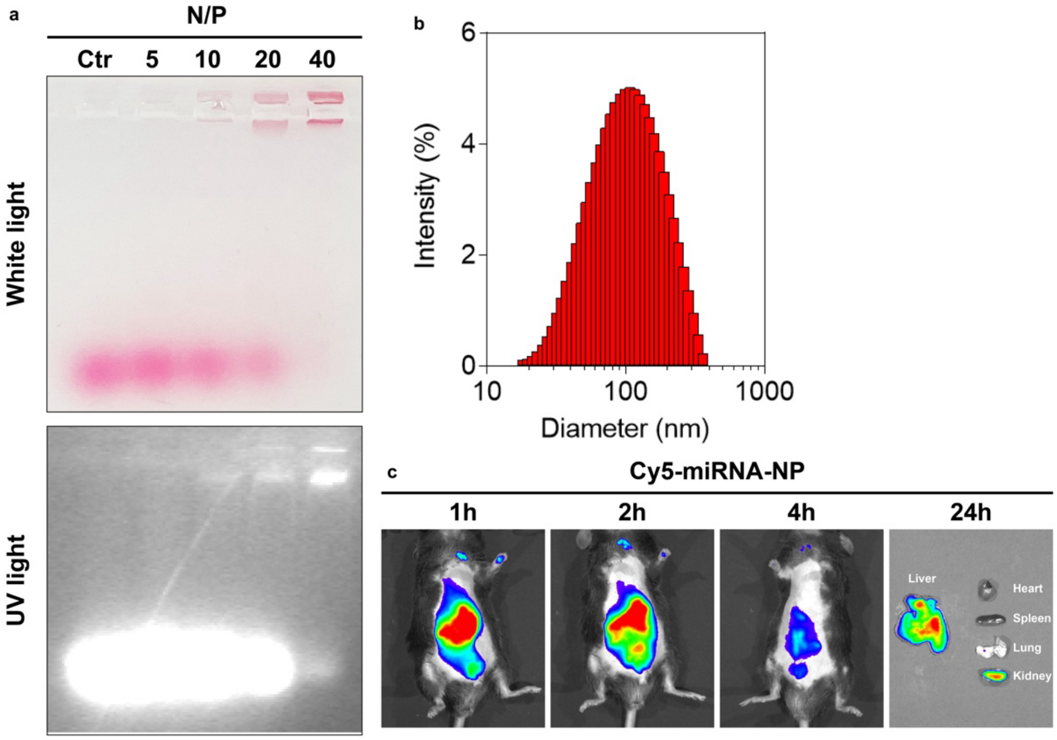

Supplementary Figure 12 Validation of *in vivo* and *in vitro* characterization of NPs. a Gel electrophoretic imaging of the binary complex of miR-28-5p antagomir /protamine with variable N:P ratios. **b** Particle size of binary complex of miR-28-5p antagomir /protamine incubated with BSA (*n* = 3 independent experiments per group). **c** *In vivo* imaging of the mice receiving intravenous injection of nano particles. *Ex vivo* imaging of different tissues collected from the mice at 24 h post injection of nano particles. Source data are provided in the Source Data file.

Supplementary Tables

Supplementary Table 1. Key resources table

REAGENT or RESOURCE	SOURCE	IDENTIFIER
Chemicals, peptides, and recombinant proteins		
Cyclopamine	MedChemExpress	HY-17024
SAG	Targetmol	T1779
17AAG	CSNpharm	CSN17209
DAPI	Beyotime	C1006

Recombinant Murine Shh	PeproTech	315-22
Recombinant Murine M-CSF	PeproTech	315-02
RNA-easy Isolation Reagent	Vazyme	R701-01
HiScript Q RT SuperMix	Vazyme	R122-01
AceQ qPCR SYBR Green Master Mix	Vazyme	Q111-02/03
High fat, fructose and cholesterol diet	Research Diets	D09100310
Collagenase D	Sigma	11088866001
DMEM	Gibco	11965092
Fetal bovine serum	Gibco	10099141C
Critical commercial assays		
BCA kit	Beyotime	P0012
Triglyceride Assay Kit	Jiancheng Bioengineering Insitute	A110-1-1
Total Cholesterol Assay Kit	Jiancheng Bioengineering Insitute	A111-1-1
LDL-c Assay Kit	Jiancheng Bioengineering Insitute	A113-1-1
HDL-c Assay Kit	Jiancheng Bioengineering Insitute	A112-1-1
ALT Assay Kit	Jiancheng Bioengineering Insitute	C009-2-1
AST Assay Kit	Jiancheng Bioengineering Insitute	C010-2-1
Mouse SHH ELISA Kit	CUSABIO	CSB-EL021266MO
Human SHH ELISA Kit	CUSABIO	CSB-E12005h
Mouse IL-1β ELISA Kit	CUSABIO	CSB-E08054m
Lipofectamine 3000	Thermo fisher scientific	L3000001
Lipofectamine-iMAX	Thermo fisher scientific	13778075
SimpleChIP® Plus Sonication Chromatin IP Kit	Cell Signaling Technology	56383
EMSA Kit	Beyotime	GS009
percoll	Biosharp	BS909
Experimental models: Organisms/strains		
Hepatocellular *Hsp90ab1* KO mice	This paper	N/A
C57BL/6 mice	Vital River	N/A
Oligonucleotides		
Primers for RT PCR	This paper	Supplementary Table 3
miR-28-5P RT-PCR primer	Genepharma	E17002
Software		
Illustrator	Adobe	http://www.adobe.com

Photoshop	Adobe	http://www.adobe.com
GraphPad Prism 8.0.2 software	GraphPad Software. Inc.	http://www.graphpad.com/ scientificsoftware/prism/

Supplementary Table 2. Antibody list

Antibody	Host Species	Type	Company name	Catalog number	Dilution
anti-Mouse	Goat	Secondary, HRP	Beyotime	A0216	1:1000
anti-Rabbit	Goat	Secondary, HRP	Beyotime	A0208	1:1000
anti-Rabbit, Dylight 555	Donkey	Secondary, Flourescence	Beyotime	A0453	1:500
Akt	Rabbit	Primary	Cell Signaling Technology (CST)	4685	1:1000
Beta Actin	Mouse	Primary	Proteintech	66009-1-Ig	1:50000
Beta Tubulin	Rabbit	Primary	Proteintech	10094-1-AP	1:5000
BMAL1	Rabbit	Primary	CST	14020	1:1000
Calnexin	Rabbit	Primary	Proteintech	10427-2-AP	1:4000
CD63	Rabbit	Primary	Abcam	ab217345	1:1000
EN2	Rabbit	Primary	ABclonal	A17480	1:1000
F4/80	Rabbit	Primary	Servicebio	GB113373-100	1:1000
FOXJ3	Rabbit	Primary	Proteintech	19751-1-AP	1:1000
GAPDH	Mouse	Primary	Proteintech	60004-1-Ig	1:100000
GLI1	Rabbit	Primary	Proteintech	66905-1-Ig	1:10000
GPM6A	Rabbit	Primary	Proteintech	15044-1-AP	1:5000
HA	Rabbit	Primary	Abcam	ab9110	1:5000
Hsp70	Rabbit	Primary	Abcam	ab2787	1:1000
Hsp90 alpha	Rabbit	Primary	Abcam	ab2928	1:1000
Hsp90 beta	Rabbit	Primary	Abcam	ab2927	1:5000
IKKβ	Rabbit	Primary	CST	2370	1:1000
IκBα	Rabbit	Primary	CST	9242	1:1000
Myc	Mouse	Primary	CST	2276	1:1000
NF-kB p65	Rabbit	Primary	Abcam	ab16502	1:1000
NF-kB p65 (phospho S536)	Rabbit	Primary	Abcam	ab76302	1:1000
PCNA	Mouse	Primary	Beyotime	AF0261	1:1000
Phospho-Akt (Ser473)	Rabbit	Primary	CST	5012	1:1000
Phospho-IKKα/β (Ser176/180)	Rabbit	Primary	CST	2697	1:1000
Phospho-IκBα (Ser32/36)	Mouse	Primary	CST	9246	1:1000

RAP1B	Rabbit	Primary	Proteintech	10840-1-AP	1:1000
SHH	Rabbit	Primary	CST	2207	1:1000
SSRP1	Rabbit	Primary	Proteintech	15696-1-AP	1:1000
SUFU	Rabbit	Primary	Proteintech	26759-1-AP	1:2000
TNS3	Rabbit	Primary	Proteintech	20053-1-AP	1:1000
TSG101	Rabbit	Primary	Abcam	ab125011	1:5000
Ubiquitin	Mouse	Primary	Santa Cruz Biotechnology	sc-8017	1:1000

Supplementary Table 3. Oligo list

Primers for qRT-PCR			
Species	Gene	Forward	Reverse
Mus musculus	Srebf1	GCAGCCACCATCTAGCCTG	CAGCAGTGAGTCTGCCTTGAT
	Srebf2	CAGGTGCAGACGGTACAGG	CGACCCTTACTGGCACTTGAA
	Fasn	GGAGGTGGTGATAGCCGGTAT	TGGGTAATCCATAGAGCCCAG
	Hmgcs	AACTGGTGCAGAAATCTCTAGC	GGTTGAATAGCTCAGAACTAGCC
	Il-1β	GCAACTGTTCCTGAACTCAACT	ATCTTTTGGGGTCCGTCAACT
	Tnf-α	GCCTCTTCTCATTCCTGCTT	TGGGAACTTCTCATCCCTTTG
	Il-6	TAGTCCTTCCTACCCCAATTTCC	TTGGTCCTTAGCCACTCCTTC
	Col1α1	GCTCCTCTTAGGGGCCACT	CCACGTCTCACCATTGGGG
	Col3α1	CTGTAACATGGAAACTGGGGAAA	CCATAGCTGAACTGAAAACCACC
	α-Sma	CCCAGACATCAGGGAGTAATGG	TCTATCGGATACTTCAGCGTCA
	Smo	TTGTGCTCATCACCTTCA GC	TGGCTTGGCATAGCACATAG
	Usp31	GAGCAAAGCAGATTCTTCCAGG	CTCAAGCTCCGGTCAGAAGTC
	Gli1	CTACTCGGGGTTCAATGATGC	TGTGGAGTTGGGGCTAGACAT
	Hsp90aa1	TGTTGCGGTACTACACATCTGC	GTCCTTGGTCTCACCTGTGATA
	Hsp90ab1	GTCCGCCGTGTGTTCATCAT	GCACTTCTTGACGATGTTCTTGC
	Bmal1	TGACCCTCATGGAAGGTTAGAA	GGACATTGCATTGCATGTTGG
	Foxo1	CCCAGGCCGGAGTTTAACC	GTTGCTCATAAAGTCGGTGCT
	Klf1	GGCGAACTTTGGCACCTAAGA	AGAAGGGACGATGTCCAGTGT
	Klf2	CAGCGCCCTTGAGAACAGAAT	GTGGACGTTTGGAGACCCTTG
	Lhx1	TTCCTCTTGAACGTGTTGGAC	TCGGTCAGGTTGCATTTACATT
	Mitf	CAAATGGCAAATACGTTACCCG	CTCCCTTTTTATGTTGGGAAGGT
	Sp1	GCCGCCTTTTCTCAGACTC	TTGGGTGACTCAATTCTGCTG
	Tgfbr1	TCTGCATTGCACTTATGCTGA	AAAGGGCGATCTAGTGATGGA
	Timp1	GCAACTCGGACCTGGTCATAA	CGGCCCGTGATGAGAAACT
Homo sapiens	HSP90AA1	GCTTGACCAATGACTGGGAAG	AGCTCCTCACAGTTATCCATGA
	HSP90AB1	CATCTCCATGATTGGGCAGTT	CTTTGACCCGCCTCTCTTCTA
	GAPDH	CTGGGCTACACTGAGCACC	AAGTGGTCGTTGAGGGCAATG
Oligo sequences for siRNA			
Species	Gene	Forward	Reverse
	Bmal1	AGCAAACUACAAGCCAACATT	UGUUGGCUUGUAGUUUGCUTT

Mus musculus	Foxo1	GCAACGAUGACUUUGAUAATT	UUAUCAAAGUCAUCGUUGCTT
	Klf1	GGCGAACUUUGGCACCUAATT	UUAGGUGCCAAAGUUCGCCTT
	Klf12	GACCUUAGAUAGCGUUAAUTT	AUUAACGCUAUCUAAGGUCTT
	Lhx1	GGUACCAAAUGCGCCGGUUTT	AACCGGCGCAUUUGGUACCTT
	Mitf	GACGGUACCAUCACCUUUATT	UAAAGGUGAUGGUACCGUCTT
	Smo	GCAAGAUCAACGAGACCAUTT	AUGGUCUCGUUGAUCUUGCTT
	Sp1	GCCCUUAUUACCACCAAUATT	UAUUGGUGGUAAUAAGGGCTT
	Srebf1	CCAGCUCAUCAACAACCAATT	UUGGUUGUUGAUGAGCUGGTT
	Usp31	CCACAAACCUGCACUUUAUTT	AUAAAGUGCAGGUUUGUGGTT
Oligo sequences for miRNA mimicus and antagomirs			
Species	Gene	Forward	Reverse
Mus musculus	miR-NC mimic	UUCUCCGAACGUGUCACGUTT	ACGUGACACGUUCGGAGAATT
	miR-28-5p mimic	AAGGAGCUCACAGUCUAUUGAG	CAAUAGACUGUGAGCUCCUUUU
	antagomiR-NC	CAGUACUUUUGUGUAGUACAA	
	mir-28-5p antagomiR	CUCAAUAGACUGUGAGCUCCUU	
