# Supplementary material for: Sonic hedgehog-heat shock protein 90β axis promotes the development of nonalcoholic steatohepatitis in mice
Source: Nat Commun. 2024 Feb 12;15:1280. doi: 10.1038/s41467-024-45520-8 (PMC10859387; doi:10.1038/s41467-024-45520-8)
Supplement: Supplementary file 3 — Reporting Summary [file 41467_2024_45520_MOESM3_ESM.pdf]

nature portfolio

Corresponding author(s): Xiaojun Xu

Last updated by author(s): Jan 10, 2024

Reporting Summary

Nature Portfolio wishes to improve the reproducibility of the work that we publish. This form provides structure for consistency and transparency in reporting. For further information on Nature Portfolio policies, see our Editorial Policies and the Editorial Policy Checklist.

Statistics

For all statistical analyses, confirm that the following items are present in the figure legend, table legend, main text, or Methods section.

n/a	Confirmed	
☐	☒	The exact sample size (*n*) for each experimental group/condition, given as a discrete number and unit of measurement
☐	☒	A statement on whether measurements were taken from distinct samples or whether the same sample was measured repeatedly
☐	☒	The statistical test(s) used AND whether they are one- or two-sided<br>*Only common tests should be described solely by name; describe more complex techniques in the Methods section.*
☒	☐	A description of all covariates tested
☐	☒	A description of any assumptions or corrections, such as tests of normality and adjustment for multiple comparisons
☐	☒	A full description of the statistical parameters including central tendency (e.g. means) or other basic estimates (e.g. regression coefficient) AND variation (e.g. standard deviation) or associated estimates of uncertainty (e.g. confidence intervals)
☐	☒	For null hypothesis testing, the test statistic (e.g. *F*, *t*, *r*) with confidence intervals, effect sizes, degrees of freedom and *P* value noted<br>*Give P values as exact values whenever suitable.*
☒	☐	For Bayesian analysis, information on the choice of priors and Markov chain Monte Carlo settings
☒	☐	For hierarchical and complex designs, identification of the appropriate level for tests and full reporting of outcomes
☒	☐	Estimates of effect sizes (e.g. Cohen's *d*, Pearson's *r*), indicating how they were calculated

Our web collection on statistics for biologists contains articles on many of the points above.

Software and code

Policy information about availability of computer code

Data collection	RNA-seq, TargetScan, miRDB, ABI 7500 Real-Time PCR System, Olympus Confocal FV3000, Endnote X9, Western images by chemiluminescence using Tanon 4600.
Data analysis	Graphpad Prism 8.0.2 was used for data analysis.

For manuscripts utilizing custom algorithms or software that are central to the research but not yet described in published literature, software must be made available to editors and reviewers. We strongly encourage code deposition in a community repository (e.g. GitHub). See the Nature Portfolio guidelines for submitting code & software for further information.

Data

Policy information about availability of data

All manuscripts must include a data availability statement. This statement should provide the following information, where applicable:

- Accession codes, unique identifiers, or web links for publicly available datasets
- A description of any restrictions on data availability
- For clinical datasets or third party data, please ensure that the statement adheres to our policy

The raw RNA-seq data are available for download from NCBI Gene Expression Omnibus under the accession numbers GSE232758 and GSE232759. There are no restrictions on data availability in the current work. Source data are provided with this paper.

Research involving human participants, their data, or biological material

Policy information about studies with human participants or human data. See also policy information about sex, gender (identity/presentation), and sexual orientation and race, ethnicity and racism.

Reporting on sex and gender	Sex and gender were not considered in our study design.
Reporting on race, ethnicity, or other socially relevant groupings	N/A
Population characteristics	The liver samples from patients are obtained from 5 healthy donors, 14 NAFL patients, 12 NASH patients. The serum samples were obtained from 19 healthy donors and 19 NASH patients.
Recruitment	Human blood specimens were collected at multiple centers by qualified medical staff. Nine individuals with NASH and 19 controls without NASH were enrolled without any age or sex preference. We excluded individuals with known blood-transmitted infectious diseases, chronic inflammatory systemic diseases, and other significant disease like severe cardiac, liver, or kidney diseases or tumors. Human liver samples from patients with NAFLD were diagnosed by abdominal ultrasound and verified for liver histology. The liver samples were from treatment-naïve patients following bariatric surgery to rule out the experimental complications due to medication. Normal human liver tissue comprised the uninvolved surrounding tissue and was obtained from NAFLD-free donors undergoing partial hepatectomy for hepatocarcinoma.<br>Information obtained from all participants and/or their relatives before sample collection was kept confidential. Informed consents were obtained from all participants and the experiments conformed to the principles outlined in the WMA Declaration of Helsinki and the Department of Health and Human Services Belmont Report. All participants received compensation.
Ethics oversight	All procedures were approved by the Ethical Committee of Guangdong Provincial People's Hospital (Guangdong Academy of Medical Sciences) and the Beijing You'An Hospital.

Note that full information on the approval of the study protocol must also be provided in the manuscript.

Field-specific reporting

Please select the one below that is the best fit for your research. If you are not sure, read the appropriate sections before making your selection.

☒ Life sciences ☐ Behavioural & social sciences ☐ Ecological, evolutionary & environmental sciences

For a reference copy of the document with all sections, see nature.com/documents/nr-reporting-summary-flat.pdf

Life sciences study design

All studies must disclose on these points even when the disclosure is negative.

Sample size	Sample size of each experiment is indicated in the figure legends. Sample sizes were based on the experience of the authors with molecular and invivo studies as published in many studies. For animal models, experiments were designed to detect differences between treatment groups or genotype-dependent effects. Sample sizes may vary depending on animal availability. Sample size for cell based assays were determined based on sample availability.
Data exclusions	No exclusion of data was made.
Replication	All experimental data was reliably reproduced in multiple independent experiments as indicated in the figure legends.The replication number for each experiment is indicated in the legend of the corresponding figure.
Randomization	For in vitro studies, the cells from each cell line required for all tested conditions were pooled, equal number of cell were then seeded and stimulated/treated randomly. For in vivo studies, the animal were allocated to experimental groups to ensure equal litter/sex/age across groups.
Blinding	For in vitro experiments, investigators were not blinded to group allocation during data collection and analysis. For in vivo experiments, treatments/genotypes were not disclosed to investigators generating quantitative readouts during data collection but investigators were not blinded during data analysis.

Reporting for specific materials, systems and methods

We require information from authors about some types of materials, experimental systems and methods used in many studies. Here, indicate whether each material, system or method listed is relevant to your study. If you are not sure if a list item applies to your research, read the appropriate section before selecting a response.

Materials & experimental systems

n/a	Involved in the study
☐	☒ Antibodies
☐	☒ Eukaryotic cell lines
☒	☐ Palaeontology and archaeology
☐	☒ Animals and other organisms
☒	☐ Clinical data
☒	☐ Dual use research of concern
☒	☐ Plants

Methods

n/a	Involved in the study
☒	☐ ChIP-seq
☒	☐ Flow cytometry
☒	☐ MRI-based neuroimaging

Antibodies

Antibodies used

The antibodies information is provided in Supplementary Table 2.
Antibody Host Species Type Company name Catalog number Dilution
anti-Mouse Goat Secondary, HRP Beyotime A0216
1:1000
anti-Rabbit Goat Secondary, HRP Beyotime A0208 1:1000
anti-Rabbit, Dylight 555 Donkey Secondary, Flourescence Beyotime
A0453 1:500
Akt Rabbit Primary Cell Signaling Technology (CST) 4685 1:1000
Beta Actin Mouse Primary Proteintech 66009-1-Ig
1:50000
Beta Tubulin Rabbit Primary Proteintech 10094-1-AP
1:5000
BMAL1 Rabbit Primary CST 14020 1:1000
Calnexin Rabbit Primary Proteintech 10427-2-AP
1:4000
CD63 Rabbit Primary Abcam ab217345 1:1000
EN2 Rabbit Primary ABclonal A17480 1:1000
F4/80 Rabbit Primary Servicebio GB113373-100 1:1000
FOXJ3 Rabbit Primary Proteintech 19751-1-AP
1:1000
GAPDH Mouse Primary Proteintech 60004-1-Ig 1:100000
GLI1 Rabbit Primary Proteintech 66905-1-Ig
1:10000
GPM6A Rabbit Primary Proteintech 15044-1-AP
1:5000
HA Rabbit Primary Abcam ab9110 1:5000
Hsp70 Rabbit Primary Abcam ab2787 1:1000
Hsp90 alpha Rabbit Primary Abcam ab2928 1:1000
Hsp90 beta Rabbit Primary Abcam ab2927 1:5000
IKKβ Rabbit Primary CST 2370 1:1000
IκBα Rabbit Primary CST 9242 1:1000
Myc Mouse Primary CST 2276 1:1000
NF-kB p65 Rabbit Primary Abcam ab16502 1:1000
NF-kB p65 (phospho S536) Rabbit Primary Abcam ab76302
1:1000
PCNA Mouse Primary Beyotime AF0261 1:1000
Phospho-Akt (Ser473) Rabbit Primary CST 5012 1:1000
Phospho-IKKα/β (Ser176/180) Rabbit Primary CST 2697
1:1000
Phospho-IκBα (Ser32/36) Mouse Primary CST 9246
1:1000
RAP1B Rabbit Primary Proteintech 10840-1-AP
1:1000
SHH Rabbit Primary CST 2207 1:1000
SSRP1 Rabbit Primary Proteintech 15696-1-AP
1:1000
SUFU Rabbit Primary Proteintech 26759-1-AP
1:2000
TNS3 Rabbit Primary Proteintech 20053-1-AP
1:1000
TSG101 Rabbit Primary Abcam ab125011 1:5000
Ubiquitin Mouse Primary Santa Cruz Biotechnology sc-8017
1:1000

Validation

All antibodies used are commercially available and validated by the manufacturers, as indicated on the respective websites of each commercial vendor. Please refer to the commercial website of each primary antibody for more details.

Eukaryotic cell lines

Policy information about cell lines and Sex and Gender in Research

Cell line source(s)	The Hepa 1-6, HepG2, and HEK293T cell lines were obtained from Cell Bank of the Chinese Academy of Sciences (Shanghai, China). All these cell lines have been provided in "Cell Culture" section.
Authentication	All cell lines have been verified by morphology check under microscopy and short tandem-repeat DNA profiling before the study.
Mycoplasma contamination	The Hepa 1-6, HepG2, and HEK293T cell lines were used immediately after being received from the Type Culture Collection of the Chinese Academy of Sciences (Shanghai, China) and certified as Mycoplasma free.
Commonly misidentified lines (See ICLAC register)	No commonly misidentified cell lines were used in this study.

Animals and other research organisms

Policy information about studies involving animals; ARRIVE guidelines recommended for reporting animal research, and Sex and Gender in Research

Laboratory animals	Male C57BL/6J mice were purchased from Beijing Vital River Laboratory Animal Technology (Beijing, China) and housed in a specific-pathogen-free facility. All mice used herein were maintained in a temperature- (22 ± 2 °C) and humidity-controlled (40-70%) animal facility under a 12 h/12 h light/dark cycle and allowed free access to food and water. As shown in Fig. 1a, the mice were fed a high fat, high fructose, and high cholesterol diet (HFFC containing 40 kcal% fat (mostly palm oil), 20 kcal% fructose, and 2% cholesterol; #D09100310, Research Diets) or a normal chow diet for 16 weeks with ad libitum access. Subsequently, vehicle or cyclopamine (20 mg/kg) was intraperitoneally injected every 2 days for 9 weeks as indicated.<br>To evaluate the effects of 17AAG, mice were fed HFFC or a normal chow diet ad libitum for 16 weeks (Supplementary Fig. 4a), following which, vehicle or 17AAG (1 mg/kg) was every 2 days for 9 weeks to the respective groups.<br>Hsp90β-flox mice were generated by Viewsolid Biotech using the CRISPR-cas9 system. sgRNA-directed cas9 endonuclease cleavage inserted LoxP sites at both ends of exons 1-3 by homologous recombination (Supplementary Fig. 2a). Hsp90β-flox mice were then mated with Albumin-Cre mice to produce the Hsp90β fl/fl; Alb-cre+/wt (Hsp90βΔHep) mice (Supplementary Fig. 2a-c). The 8-weeks old Hsp90β fl/fl or Hsp90βΔHep mice were fed chow or HFFC diet for 33 weeks. At the 9th week, mice were injected with ADV-NC or ADV-Shh 1.0 × 109 pfu per mouse through their tail veins and fed HFFC diet for an additional 9 weeks (Fig. 3a).<br>To evaluate the therapeutic effects of miRNA inhibitors, mice were fed chow or HFFC diet for 8 weeks. Subsequently, nanoparticles encapsulating miR-NC or miR-28-5p antagomir were intravenously injected (5 nmol/mouse, twice a week) for 4 weeks.
Wild animals	This study did not involve wild animals.
Reporting on sex	Only male mice participated in this experimental study.
Field-collected samples	This study did not involve samples collected from the field.
Ethics oversight	The cervical dislocation was used for mice euthanasia. All animal care and related experiments outlined in this study were performed in accordance with the Guidelines for the Care and Use of Laboratory Animals drafted by the US National Institutes of Health (NIH Publication, 8th Edition, 2011). Animal experiments were conducted following the Guidelines for Animal Experimentation of China Pharmaceutical University, and the protocols were approved by the Science and Technology Department of Jiangsu Province (SYXK (SU) 2016-0011).

Note that full information on the approval of the study protocol must also be provided in the manuscript.

Plants

Seed stocks	N/A
Novel plant genotypes	N/A
Authentication	N/A
